# Non-Targeted Metabolomics Profiling and Anti-Inflammatory Potential of Star Anise Extract in Rats with Cold Stress—Aggravated Acute Lung Injury

**DOI:** 10.3390/metabo16070486

**Published:** 2026-07-10

**Authors:** Mengli Zhang, Min Ou, Xuancheng Wang, Song Kou, Xianghua Xia, Wenyan Fan, Senhua Lu, Yu Chen, Xiaonan Yang

**Affiliations:** 1 National Engineering Research Center for the Development of Southwestern Endangered Medicinal Materials, Guangxi Botanical Garden of Medicinal Plants, Nanning 530023, China; 2 Yulin Center for Food and Drug Control, Yulin 537000, China

**Keywords:** acute lung injury, cold stress, star anise, inflammation, metabolomics

## Abstract

**Background/Objectives:** This study is the first to investigate the potential mechanism of star anise extract (SAE) in protecting against cold stress-aggravated acute lung injury (CSALI) in rats. **Methods:** A rat CSALI model was induced via combined lipopolysaccharide challenge and cold stress exposure. The preventive effects of SAE were evaluated using cytotoxicity assays, quantification of biochemical indices and inflammatory factors, and histopathological examination. Ultra-performance liquid chromatography coupled with high-resolution mass spectrometry (UPLC–HRMS)-based serum metabolomics was employed to systematically profile CSALI-associated metabolic alterations and decipher the potential mechanism underlying the preventive effects of SAE. **Results:** SAE alleviated pathological progression of CSALI, suppressed inflammatory cell migration, markedly reduced pulmonary inflammatory cell infiltration, and ameliorated lung tissue injury in CSALI rats. SAE also improved abnormal liver function indicators and lowered the levels of pro-inflammatory factors in both serum and bronchoalveolar lavage fluid (BALF). Serum metabolomics analysis identified and annotated 24 disease-altered differential metabolites and evaluated the protective effects of SAE on them. These metabolites were significantly enriched in two key metabolic pathways related to the pathogenesis of CSALI, including arachidonic acid metabolism and glycerophospholipid metabolism. Furthermore, based on metabolite changes, phospholipase A2 was hypothesized as a potential key regulatory factor that may cooperate with arachidonic metabolism to suppress the inflammatory cascade. **Conclusions:** These findings demonstrated that SAE exerted prominent anti-inflammatory activity and effectively protected against lung injury in CSALI rats.

## 1. Introduction

Ethnic medicine constitutes an integral component of the treasured heritage of traditional Chinese medicine (TCM). Star anise (SA), the dried mature fruit of the Schisandraceae plant *Illicium verum* Hook. F. is officially listed in the Chinese Pharmacopoeia. Its medicinal use dates back to the Tang Dynasty, with the earliest written record documented in the Book of Herbal Pictures [1]. Traditionally, it is utilized to warm yang, dissipate cold, regulate qi, and relieve pain [2]. The phytochemical profile of SA mainly includes volatile oils (primarily trans-anethole, accounting for 80–90%), shikimic acid, flavonoids, and organic acids [3,4]. Pharmacological studies indicated that star anise extract (SAE) reduced airway inflammation by enhancing Foxp3 regulatory T cells and inhibiting Th2-type cytokines [5]. Furthermore, in a rheumatoid arthritis (RA) model, SAE modulated human RA fibroblast-like synovial cells (RA-FLS) by regulating lipid and amino acid metabolism pathways, suggesting its therapeutic potential for inflammatory autoimmune diseases [6].

Endotoxin (lipopolysaccharide, LPS)-induced pneumonia, a common clinical respiratory disorder triggered by Gram-negative bacteria, involves complex pathophysiological processes including oxidative stress, dysregulated inflammatory responses, and immune dysfunction. During cold seasons, both the incidence and severity of respiratory infections increase [7]. In recent years, climate changes, including extreme high or low temperatures, have been shown to correlate with a higher prevalence of community-acquired pneumonia [8]. The synergistic interaction between LPS stimulation and cold stress is a key trigger for the initiation and progression of lung inflammation under such conditions [9]. At the mechanistic level, cold stress directly compromises the integrity of the lung epithelial barrier and impairs mucosal defense, thereby facilitating endotoxin adhesion and invasion. Concurrently, it induces oxidative stress, aberrant immune cell activation, and stimulation of the TLR4 and NF-κB signaling pathways [10,11,12,13], thereby creating a permissive microenvironment for endotoxin-mediated inflammation. Given that LPS itself acts as a potent agonist of the TLR4/NF-κB pathway [14], its combination with cold stress results in dual activation of this pathway. This synergism drives M1 polarization of pulmonary macrophages, massive release of pro-inflammatory cytokines, and amplification of the pulmonary inflammatory cascade [14,15]. In this study, we established a rat model using a combination of low-temperature exposure and LPS challenge. While low temperature alone does not directly induce lung injury, it synergizes with LPS to activate the aforementioned pathological cascade. Recent studies have confirmed that hyperactivation of the TLR4/NF-κB pathway and imbalanced macrophage polarization are core pathological events in acute lung injury induced by superimposed cold stress and LPS [16,17]. Sustained activation of this signaling axis can culminate in uncontrolled pulmonary inflammation, ultimately leading to irreversible structural damage, including pulmonary congestion, edema, and fibrosis.

Despite the considerable promise of SA-based product development, substantial challenges remain. A pivotal issue in advancing modern TCM products lies in the integration of TCM theories—such as “warming yang and dispelling cold”—with contemporary pharmacological discoveries [18]. Given that aromatic and pungent TCMs often function as full or partial agonists of thermosensitive transient receptor potential (TRP) channels [19,20], and considering that SA is a prototypical warming medicinal herb, we hypothesize that SA may likewise belong to this category of TCMs. Notably, systematic metabolomic research exploring the regulatory effects of SA under acute cold stress conditions is essential to verify this hypothesis [21]. As a pivotal branch of systems biology, metabolomics enables sensitive detection of endogenous metabolite alterations and accurate identification of metabolic disorders associated with disease pathogenesis and drug efficacy. Therefore, in this study, we prepared SAE via ultrasonic-assisted ethanol extraction and established a rat model of cold stress-aggravated acute lung injury (CSALI) induced by LPS and cold stimulation. We comprehensively evaluated the anti-inflammatory effects and underlying mechanisms of SAE through integrated in vitro and in vivo experiments. UPLC–MS/MS-based non-targeted metabolomics was employed to identify disease-altered differential metabolites and characterize the preventive effects of SAE in CSALI rats. These findings elucidate the anti-inflammatory properties and potential metabolic regulatory mechanisms of SAE, characterize the metabolic features of CSALI, and provide robust experimental evidence and a theoretical basis for the development of SAE as a natural product for CSALI.

## 2. Materials and Methods

### 2.1. Chemicals and Reagents

LPS (Amsterdam, the NetherlandsL4391) was purchased from Sigma-Aldrich (St Louis, MO, USA). Hematoxylin and eosin (H&E) staining kit, and 4% paraformaldehyde fixative (batch no. C0105S, P0099) were obtained from Shanghai Beyotime Biotechnology Co., Ltd. (Shanghai, China). Assay kits for alanine aminotransferase (ALT), aspartate aminotransferase (AST), alkaline phosphatase (ALP), total bilirubin (TBIL), and direct bilirubin (DBIL) were purchased from Shanghai Kehua Bio-engineering Co., Ltd. (Shanghai, China). ELISA kits for transforming growth factor-β (TGF-β) and interleukin-6 (IL-6) (batch no. 202409260601) were purchased from Zhanxin Biotechnology Co., Ltd. (Quanzhou, China). HPLC-grade methanol and acetonitrile were obtained from Thermo Fisher Scientific (Waltham, MA, USA), and HPLC-grade formic acid was obtained from Merck (Darmstadt, Germany).

### 2.2. Cells and Animals

The mouse monocyte macrophage RAW264.7 cells were obtained from the Cell Center, Institute of Basic Medical Sciences, Chinese Academy of Medical Sciences. SPF-grade male SD rats (8–9 weeks, 200–220 g) were purchased from Changsha Tianqin Biotechnology Co., Ltd. (Certificate SCXK (Changsha, China) 20240143). Rats were housed under controlled environmental conditions: temperature of (23 ± 2) °C, relative humidity of 50%, and a 12 h light/12 h dark cycle. After one week of acclimatization, all animal experiments were conducted in strict accordance with institutional guidelines for experimental animal management (Ethical Approval No. GXBGMP-20240503).

### 2.3. Preparation of SAE

Dried fruits of SA were collected from Shanglin County, Guangxi Zhuang Autonomous Region, and the samples were stored at the Southwest National Engineering Research Center for Endangered Medicinal Materials. SAE was extracted following previously reported methods with some modifications [5]. Briefly, dried SA fruits were crushed and passed through a 40-mesh sieve. The powder was weighed and mixed with 80% ethanol with a solid-to-liquid ratio of 1:3, and the mixture was ultrasonically treated at 50 °C for 40 min. The residue was subsequently refluxed twice in 80% ethanol (30 min each), and the two filtrates were combined and concentrated under reduced pressure using a rotary vacuum evaporator. The concentrated solution was then freeze-dried using a vacuum freeze dryer and stored at −80 °C until use. The sample was considered completely dry when the weight difference between two consecutive freeze-drying cycles was less than 0.1 g, and the yield of SAE was calculated to be approximately 10.1%.

### 2.4. Cell Viability Assay

RAW264.7 cells in the logarithmic growth phase were counted, and the cell suspension was adjusted to 1 × 10^4^ cells/well and seeded into 96-well plates. After 24 h of incubation, the medium was aspirated and replaced with 100 μL/well of SAE-containing medium at final concentrations of 0.1, 1, 10, 20, 50, 100, and 200 μg/mL. Blank wells (medium without cells) and control wells (cells cultured in medium without SAE) received an equal volume of medium. After 24 and 48 h of treatment, 10 μL/well of the cell counting kit-8 (CCK-8) reagent was added to each well, and the plates were further incubated at 37 °C for 3 h. The absorbance was measured at 450 nm, and cell viability was calculated as (A_test − A_blank)/(A_control − A_blank) × 100%. Each experimental group was set up with six biological replicates.

For the LPS stimulation experiment, cells were inoculated at 5 × 10^3^ cells/well. After 24 h of incubation to allow cell attachment, the medium was replaced with fresh medium containing 1 μg/mL LPS and SAE at final concentrations of 5, 10, or 20 μg/mL. Control (without LPS stimulation) and model (with LPS stimulation but without SAE treatment) received an equal volume of medium. After 24 h, the cell viability was determined using the CCK-8 assay as described above.

### 2.5. Establishment of CSALI Model and Drug Administration

A rat model of CSALI was established according to previously published protocols [7]. The preparation of SAE was detailed in Section 2.3, and the overall experimental design is illustrated in Figure S1. After one week of acclimatization, the rats were randomly divided into the control group (C-group, n = 8) and the LPS-treated groups (n = 40). All rats were anesthetized by intraperitoneal injection of sodium pentobarbital (40 mg/Kg). Subsequently, the LPS-treated groups underwent tracheal intubation and received intratracheal instillation of LPS (5 mg/Kg, 0.2 mL), which was perfused twice at 24 h intervals. The C-group was intratracheally instilled with an equivalent volume of saline. After that, the LPS-treated rats were divided into five groups (n = 8 per group): the Model group (M-group), LPS + SAE low-dose group (BL, 0.5 g/Kg), LPS + SAE medium-dose group (BM, 1 g/Kg), LPS + SAE high-dose group (BH, 2 g/Kg), and LPS + dexamethasone group (positive control, P-group, 2 mg/Kg). The C-group and the M-group were administered equal volumes of saline. All groups were administered intragastrically once daily for 14 consecutive days. During the administration period, all LPS-treated rats (i.e., all groups excluding the C-group) were exposed to 4 °C for 4 h daily to sustain the CSALI condition.

One hour after the final administration, rats were weighed and deeply anesthetized via intraperitoneal injection of 2% pentobarbital sodium (40 mg/Kg). Loss of tail, hindlimb, and eyelid reflexes, together with slow and regular respiration, indicated complete anesthesia. Rats were subsequently euthanized by cervical dislocation. Bronchoalveolar lavage fluid (BALF) was collected from the left lung using phosphate-buffered saline (PBS, pH = 7.4). Lavage was performed three times with 1 mL of PBS each, allowing a 30 s interval between instillations. The average recovery rate of the lavage fluid was approximately 70%.

### 2.6. Serum Biochemical Analysis and Inflammatory Factor Determination

Before BALF collection, blood was obtained from the abdominal aorta and centrifuged at 4000 *g* rpm for 15 min at 4 °C. Serum was isolated and stored at −80 °C. Serum levels of ALP, ALT, AST, TBIL, and DBIL were quantified using a Hitachi automated analyzer. IL-6 and TGF-β levels in both serum and BALF were determined by ELISA according to the manufacturer’s instructions.

### 2.7. Histopathological Examination of Lung Tissue

The right upper lobe of the lung was perfused with PBS to remove residual blood and then fixed in 10% neutral-buffered formalin for 24–48 h. Fixed tissues were processed for paraffin embedding, and 3 μm-thick sections were prepared. The sections were stained with H&E and then observed under a microscope. Images of the stained sections were captured using the Leica Application Suite V4.12 (Leica Microsystems, Wetzlar, Germany).

### 2.8. Serum Metabolomics Analysis

For serum sample preparation, 300 μL of serum was mixed with 3 volumes of methanol, then centrifuged at 13,000 *g* rpm for 20 min at 4 °C, and dried under vacuum at 40 °C. The dried residues were reconstituted in 150 μL of methanol, vortexed thoroughly, and centrifuged again under the same conditions. The supernatant was used for UPLC-Q/TOF-MS analysis. Quality control (QC) samples were prepared by mixing equal aliquots (4 μL) of each sample.

The non-targeted metabolomic analysis was conducted using a Waters ACQUITY UPLC System (Waters, Milford, MA, USA), coupled with a SYNAPT G2-Si HDMS (Waters, Milford, MA, USA). LC separation was performed using an ACQUITY UPLC HSS T3 column (100 × 2.1 mm, 1.8 μm, Waters, Milford, MA, USA) maintained at 40 °C. During separation, the flow rate was set to 0.4 mL/min, and the injection volume was 2 μL. The mobile phase A was water containing 0.1% formic acid, and the mobile phase B was acetonitrile with 0.1% formic acid. The gradient elution procedure was 0–3 min, 95% A; 3–5 min, 80% A; 5–10 min, 50% A; 10–12 min, 0% A; 12.1–14 min, 95% A.

The MS data were collected using a mass spectrometer equipped with an electrospray ionization (ESI) source. The parameters were set as capillary voltage 2000 V; taper hole voltage 40 V; ion source temperature 105 °C; desolvation gas temperature 400 °C; taper hole gas flow rate 50 L/h; desolvation gas flow 800 L/h; high collision energy 20–30 V; and low collision energy 6 V. Leucine enkephalin was used for real-time mass calibration ([M + H]^+^ = 556.2771, [M − H]^−^ = 554.2615). Full scan data were acquired in MSE mode with a mass range of *m*/*z* = 100–1500 Da.

### 2.9. Statistical Analysis

All experimental data were expressed as mean ± SD and analyzed using SPSS 21.0 software. The raw metabolomics data were imported into Progenesis QI V2.0 (Waters, Milford, MA, USA) for peak picking, filtering, and alignment to construct a three-dimensional matrix including sample ID, *m*/*z*, retention time (RT), and peak intensity. The software automatically selected the optimal sample as the reference, and the alignment matching rate of other samples was set to exceed 80%. Subsequent grouping and parameter configuration were conducted accordingly. Metabolite preliminary annotation relied on precursor and fragment mass tolerance of <5 ppm and RT tolerance of 0.5 min. Adduct ions were defined as [M + H]^+^, [M + Na]^+^, and [2M + H]^+^ under ESI positive mode, and [M − H]^−^, [2M − H]^−^, [M + FA − H]^−^ under ESI negative mode. Compound identification was performed against the Human Metabolome Database (HMDB, http://www.hmdb.ca/, (accessed on 16 January 2026)) and ChemSpider (http://www.chemspider.com/, (accessed on 16 January 2026)). MS/MS spectral matching was calculated via the built-in cosine similarity algorithm, and theoretical isotope distribution patterns were further cross-checked to improve annotation reliability.

Univariate analyses were performed using one-way analysis of variance (ANOVA), followed by the least significant difference (LSD) post hoc test for pairwise multiple comparisons to control false-positive errors. An adjusted *p*-value < 0.05 was considered statistically significant. Multivariate statistical analyses were performed using SIMCA-P 14.0 (Umetrics, Umeå, Sweden), including unsupervised principal component analysis (PCA) and orthogonal partial least squares-discriminant analysis (OPLS-DA). A 200-times permutation test was conducted to prevent overfitting. The R2 (explanatory parameter) and Q2 (predictive parameter) values were used to assess the model’s predictability and interpretability. Fold change (FC) was calculated as the ratio of the average normalized abundance of each metabolite in the model group to that in the control group, and in the SAE-treated group to that in the model group. FC > 2.0 or <0.5, variable importance in the projection (VIP) > 1.00, and *p* < 0.05 were applied to select differential metabolites between the two groups. The metabolic pathways involved in the mass markers were constructed using MetaboAnalyst 6.0 (https://www.metaboanalyst.ca/, (accessed on 2 February 2026)), an online visualization tool.

## 3. Results

### 3.1. Effect of SAE on the Proliferation of RAW264.7 Cells

Table S1 presents the effect of SAE on RAW264.7 cell proliferation, as evaluated by the CCK-8 assay. After 24 h of treatment, SAE at concentrations of 10 and 20 μg/mL did not significantly affect cell viability compared to the control group (*p* > 0.05). However, exposure to 20 μg/mL SAE for 48 h resulted in significant cytotoxicity (*p* < 0.05). Despite this observed cytotoxicity at 48 h, the 20 μg/mL concentration was retained to establish a comprehensive dose gradient for exploring dose-dependent pharmacological responses. Therefore, both 10 and 20 μg/mL were selected for subsequent experiments.

### 3.2. Effect of SAE on LPS-Induced RAW264.7 Macrophages

**Figure 1:**
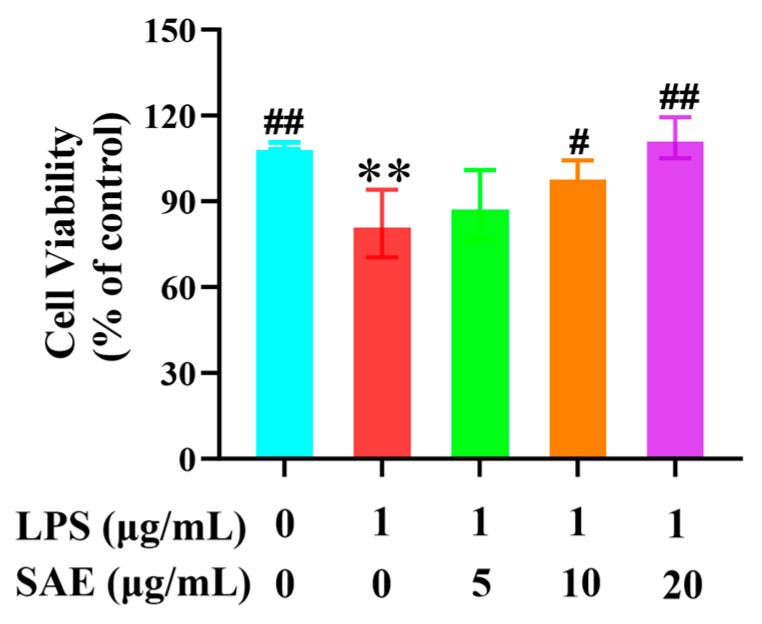
Effect of SAE on the survival rate of LPS-induced inflammatory RAW264.7 cells (mean ± SD, n = 6). ** *p* < 0.01 compared to the control group; ^#^ *p* < 0.05, ^##^ *p* < 0.01 compared to the model group. LPS, lipopolysaccharide; SAE, star anise extract.

The potential cytotoxicity of SAE toward LPS-stimulated RAW264.7 cells was assessed using the CCK-8 assay (Figure 1). At concentrations of 5, 10, and 20 μg/mL, SAE exhibited no significant cytotoxicity, with cell viability remaining above 88% throughout the treatment period.

### 3.3. Effect of SAE on Serum Liver Function Indices and Lung Histopathology

**Figure 2:**
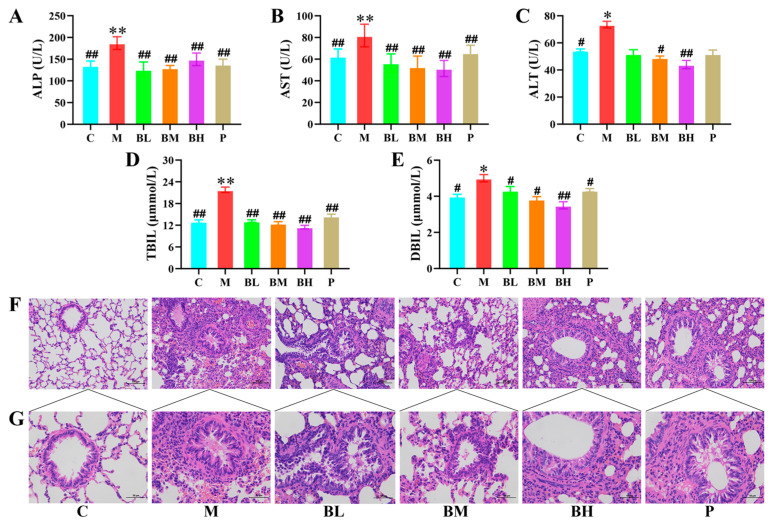
Effect of SAE on liver function indices and lung tissues. (**A**) Serum ALP levels. (**B**) Serum AST levels. (**C**) Serum ALT levels. (**D**) Serum TBIL levels. (**E**) Serum DBIL levels. (**F**) H&E staining of the lung sections (100×). (**G**) H&E staining of the lung sections (200×). (mean ± SD, n = 8). * *p* < 0.05, ** *p* < 0.01 compared to the control group; ^#^ *p* < 0.05, ^##^ *p* < 0.01 compared to the model group. ALP, alkaline phosphatase; AST, aspartate aminotransferase; ALT, alanine aminotransferase; TBIL, total bilirubin; DBIL, direct bilirubin.

The lungs and liver are functionally interconnected rather than independent organs, interacting through systemic inflammation, oxidative stress, metabolite exchange, and blood circulation [22,23]. Reports show that abnormal levels of liver function markers are nonlinearly related to the risk of chronic obstructive pulmonary disease (COPD) [22,24]. Therefore, detection of liver function markers can help uncover such mutual pathological relationships. As shown in Figure 2A–C, the serum levels of ALP, AST, and ALT in the model group were significantly elevated compared with the control group (*p* < 0.05). After SAE treatment, the serum levels of these enzymes in the SAE-treated groups were reduced to varying degrees relative to the model group (*p* < 0.05). Regarding TBIL and DBIL (Figure 2D,E), the model group exhibited significantly higher serum levels of these two indices than the control group (*p* < 0.05). Notably, SAE administration decreased serum TBIL and DBIL levels (*p* < 0.05), and the magnitude of this reduction was positively correlated with the administered SAE concentration.

H&E staining revealed distinct morphological differences among the groups (Figure 2F,G). Rats in the control group exhibited intact alveolar architecture with clear contours and no hemorrhage or inflammatory cell infiltration in the mucosa layer or airway wall. In contrast, rats in the model group displayed obvious pathological changes, including alveolar cavity enlargement, nucleus condensation (with some nuclei extruded into the alveolar cavity), airway lumen narrowing, and massive secretions accompanied by extensive inflammatory cell infiltration. The characteristic pulmonary inflammatory injury lesions observed in model rats fully confirmed the successful establishment of the CSALI model. SAE treatment alleviated inflammatory cell infiltration and significantly reduced the extent of histopathological damage compared to the model group, demonstrating that it could markedly improve pathological pulmonary lesions.

### 3.4. Effect of SAE on Inflammatory Factors in Serum and BALF

**Figure 3:**
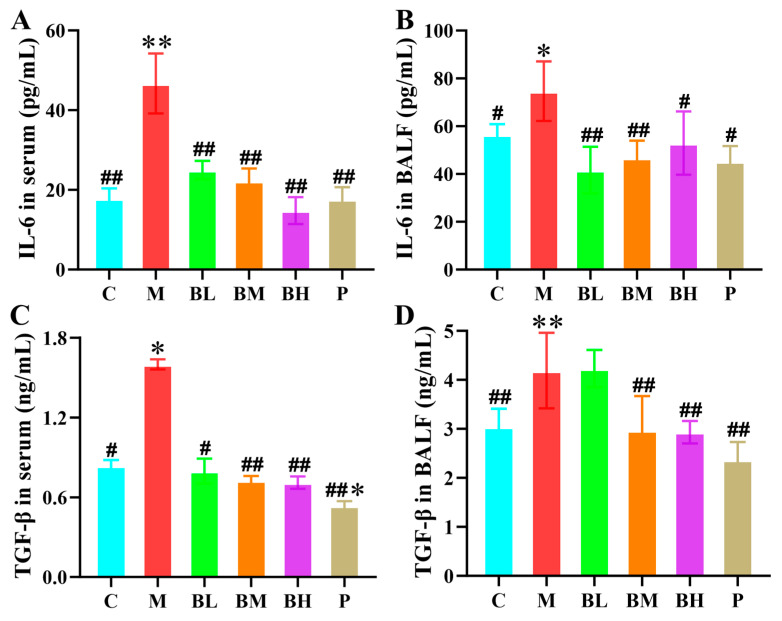
Effect of SAE on IL-6 and TGF-β levels in serum and BALF. (**A**) IL-6 levels in serum. (**B**) IL-6 levels in BALF. (**C**) TGF-β levels in serum. (**D**) TGF-β levels in BALF. (mean ± SD, n = 8). * *p* < 0.05, ** *p* < 0.01 compared to the control group; ^#^ *p* < 0.05, ^##^ *p* < 0.01 compared to the model group. IL-6, interleukin-6; TGF-β, transforming growth factor-β; BALF, bronchoalveolar lavage fluid.

As shown in Figure 3A,C, the serum levels of IL-6 and TGF-β were significantly higher in the model group than in the control group (*p* < 0.05). SAE treatment significantly reduced these serum cytokine levels compared with the model group (*p* < 0.01). In BALF, IL-6 and TGF-β levels were significantly elevated in the model group compared with the control group (*p* < 0.05), while medium and high doses of SAE treatment markedly reduced BALF IL-6 and TGF-β levels (*p* < 0.01) (Figure 3B,D). These results were consistent with the pathological changes observed in lung tissues (Figure 2F,G).

### 3.5. Metabolic Profiling Characterization

**Figure 4:**
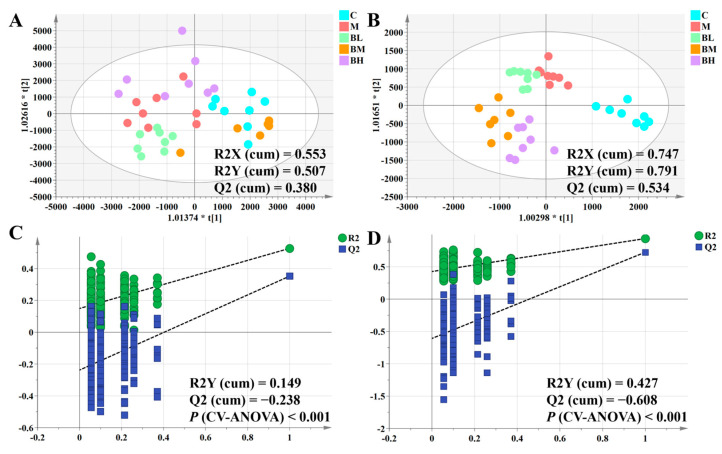
OPLS-DA analysis of metabolite profiles among five groups in both ESI modes. (**A**) OPLS-DA score plot in positive-ion mode. (**B**) OPLS-DA score plot in negative-ion mode. (**C**) Permutation test in positive-ion mode. (**D**) Permutation test OPLS-DA score plot in negative-ion mode. OPLS-DA, orthogonal partial least squares-discriminant analysis.

As shown in Figure S2, the PCA score plot revealed a clear separation between the control and model groups across both ESI modes, indicating global metabolic perturbation in the model group. In positive-ion mode, the BL group largely overlapped with the M group, suggesting negligible restoration of metabolic homeostasis. Conversely, the BM and BH groups shifted evidently toward the C group, implying a regulatory effect of SAE on metabolic profiles. However, no distinct clustering was observed in negative ion mode. Given the limitations of PCA as an unsupervised method, supervised OPLS-DA was subsequently employed to maximize class discrimination. The OPLS-DA score plots revealed distinct clustering between the control and model groups. Consistent with the PCA results, low-dose SAE exerted minimal reversal of model-induced metabolic shifts, whereas higher doses demonstrated significant regulatory activity (Figure 4A,B). Meanwhile, 200 times permutation tests were conducted to assess the reliability and applicability of the OPLS-DA model. The results indicated that the OPLS-DA model was rational and not overfit for metabolomics data analysis (Figure 4C,D).

### 3.6. Exploration of Metabolite Differences 

Based on differences in metabolite profiles, the ions with VIP > 1.00 were selected as candidate differential features. After metabolite identification, a total of 24 disease-altered differential metabolites were identified between the control and model groups, applying thresholds of FC > 2.0 or <0.5 and *p* < 0.05 (Table S2). These metabolites, significantly altered by CSALI, were further analyzed to elucidate the regulatory effects of SAE on metabolic disorders. Among them, 17 metabolites were significantly upregulated compared to the control group (*p* < 0.05), including corticosterone, indoleacrylic acid, cervonoyl ethanolamide, phosphatidylcholine (PC) (16:0/20:1(11Z)), taurochenodesoxycholic acid, cytidine, lysoPC (18:1(11Z)), n-acetylleucine, d-tryptophan, 3-succinoylpyridine, zingerone, 12,13-DHOME, lysoPE (0:0/18:0), lysoPC (18:2(9Z,12Z)), arachidonic acid, lysoPE (18:1(11Z)/0:0) and lysoPE (0:0/22:1(13Z)) (Table S2). Additionally, 7 metabolites were significantly down-regulated (*p* < 0.05), including eicosapentaenoic acid, tetracosahexaenoic acid, 15(S)-HETE, lysoPC (20:4(5Z,8Z,11Z,14Z)), lysoPE (20:0/0:0), ginkgoic acid, and sinapic acid (Table S2). They were classified as different types, including pyrimidine nucleosides, carboxylic acids and derivatives, cinnamic acids and derivatives, keto acids and derivatives, phenols, steroids and steroid derivatives, fatty acyls, glycerophospholipids, indoles and derivatives, and benzene and substituted derivatives.

**Figure 5:**
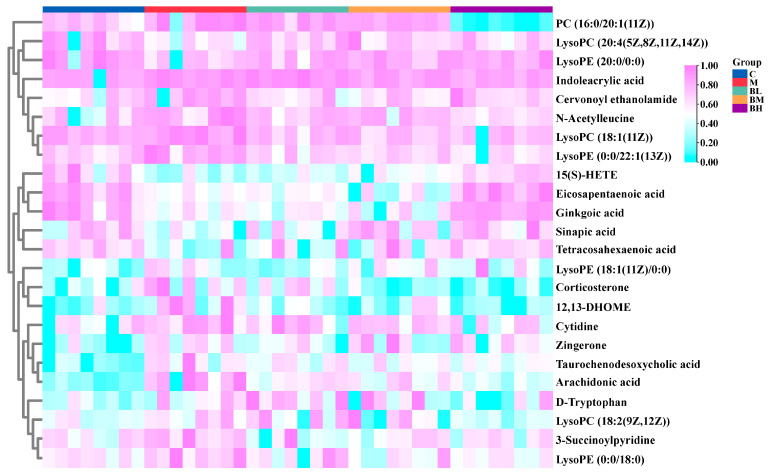
Clustering heatmap of 24 CSALI-associated differential metabolites differentiating the control and model groups. The color gradient represents the normalized relative abundance of metabolites.

A clustering heatmap was generated to visualize distinct metabolic expression patterns across experimental groups (Figure 5). Most metabolites exhibited inverse abundance profiles between the control and model groups, indicating that CSALI induced marked dysregulation of these biochemical entities. Among all SAE-treated cohorts, the BH group demonstrated the most pronounced amelioration of CSALI-induced metabolic perturbations, with metabolic signatures closely resembling those of the healthy control rats.

### 3.7. SAE Regulates Metabolic Disorders in CSALI Rats

#### 3.7.1. Pathway Analysis

**Figure 6:**
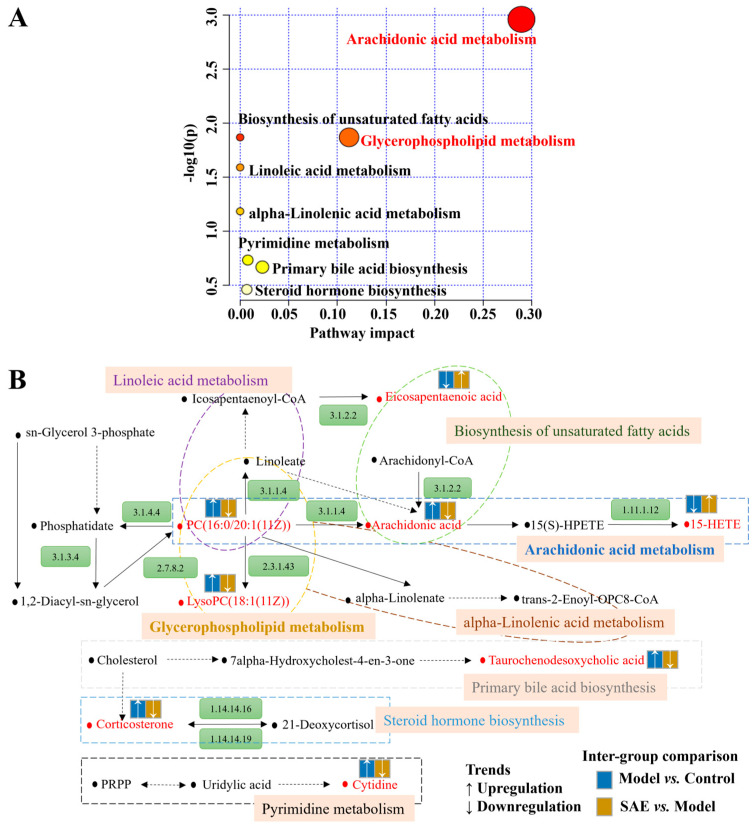
Metabolic pathway analysis of differential metabolites between the control group and the model group. (**A**) KEGG pathway analysis. (**B**) Schematic view of metabolic pathways associated with CSALI progression and SAE protective effects. The differential metabolites detected in our research were marked in red. SAE, star anise extract; KEGG, Kyoto Encyclopedia of Genes and Genomes.

Kyoto Encyclopedia of Genes and Genomes (KEGG) pathway analysis showed that the 24 differential metabolites in the serum affected 8 pathways, as shown in Figure 6A. These pathways mainly involve arachidonic acid (AA) metabolism, biosynthesis of unsaturated fatty acids, glycerophospholipid metabolism, linoleic acid metabolism, alpha-linolenic acid metabolism, pyrimidine metabolism, primary bile acid biosynthesis, and steroid hormone biosynthesis. Among them, AA metabolism (Impact = 0.2893) and glycerophospholipid metabolism (Impact = 0.1219) were the most dysregulated metabolic pathways with an impact value > 0.1. Of the 24 differential metabolites, PC (16:0/20:1(11Z)), AA, and 15-HETE were associated with AA metabolism. PC (16:0/20:1(11Z)) and lysoPC (18:1(11Z)) were associated with glycerophospholipid metabolism (Figure 6B).

#### 3.7.2. Metabolite Abundance Analysis

**Figure 7:**
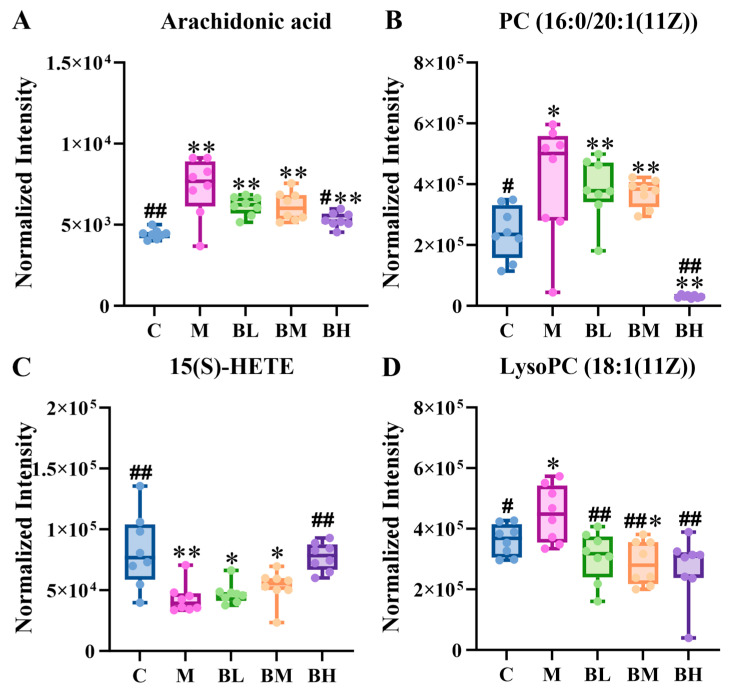
Box plots illustrating the normalized intensities of four key differential metabolites enriched in AA metabolism and glycerophospholipid metabolism pathways. (**A**) AA. (**B**) PC (16:0/20:1(11Z)). (**C**) 15(S)-HETE. (**D**) LysoPC (18:1(11Z)). (n = 8). * *p* < 0.05, ** *p* < 0.01 compared to the control group; ^#^ *p* < 0.05, ^##^ *p* < 0.01 compared to the model group. AA, arachidonic acid.

Four differential metabolites involved in AA metabolism and glycerophospholipid metabolism were further quantified, including AA, PC (16:0/20:1(11Z), 15(S)-HETE, and LysoPC (18:1(11Z)) (Figure 7). Compared with the control group, the model group exhibited significantly elevated levels of AA, PC (16:0/20:1(11Z)), and LysoPC (18:1(11Z)) (*p* < 0.05), whereas 15(S)-HETE levels were markedly decreased (*p* < 0.01). High-dose SAE administration significantly improved these abnormal metabolite levels (*p* < 0.05).

## 4. Discussion

In the present study, we established a CSALI rat model via combined low-temperature exposure and LPS challenge to evaluate the protective effects of SAE. Additionally, we assessed the anti-inflammatory activity of SAE in LPS-stimulated RAW264.7 macrophages. Histopathological examination confirmed successful model establishment, and SAE administration significantly prevented lung injury. Based on in vitro concentrations, equivalent rat doses were calculated, and a dose gradient (1-, 2-, and 4-fold) was designed. SAE treatment attenuated CSALI-induced pulmonary damage and improved the levels of inflammatory markers in both serum and BALF. Pulmonary inflammation can release inflammatory cytokines into the systemic circulation, thereby impairing hepatic synthesis of acute-phase proteins. Given that liver function indicators reflect both hepatic synthetic capacity and systemic inflammatory status, they serve as critical biomarkers in pulmonary pathologies. Consistent with this, patients with lung diseases often exhibit systemic metabolic disorders, which are frequently first reflected in hepatic enzyme and lipid metabolism profiles [25,26]. In CSALI rats, serum levels of ALP, AST, ALT, TBIL, and DBIL were significantly elevated, indicating pathological crosstalk between lung and liver. Following SAE administration, these indicators showed varying degrees of reduction in the treated group.

Serum metabolomics has become a crucial bridge connecting basic research and clinical translation. In this work, 24 key differential metabolites were identified using UPLC-MS/MS, which appear to play an important role in CSALI progression. Interestingly, SAE administration ameliorated the dysregulated profiles of these key differential metabolites (Table S2). KEGG pathway analysis revealed that they were predominantly enriched in AA metabolism and glycerophospholipid metabolism (impact value > 0.1), both of which belong to lipid metabolism. Lipids are essential cellular components, and disturbances in lipid metabolism can trigger various inflammatory responses [27]. AA metabolism and glycerophospholipid metabolism were reported as key metabolic pathways in inflammation-induced lung injury [28]. Previous studies have reported that serum concentrations of phosphatidylethanolamine (PE) and PC, as well as PC/lysophosphatidylcholine (lysoPC) and PE/lysophosphatidylethanolamine (lysoPE) ratios, were significantly increased, whereas LPC levels were significantly decreased in patients with CAP [29]. Similar alterations have been observed in pneumonia and sepsis [30,31]. Phospholipase plays an important role in these pathways (EC: 3.1.1.4; 3.1.4.4; 3.1.3.4). LPC and LPE are generated primarily through hydrolysis by phospholipase A2 (PLA2) [32,33]. A large body of evidence indicates that PLA2 is closely related to lung diseases, with possible mechanisms involving mitochondrial autophagy, synergistic effects with the AA metabolic pathway, and modulation of cell-to-cell communication and conduction [34,35,36]. LPCs exert biological effects by binding to G protein-coupled receptors and Toll-like receptors (TLRs) [37], among which TLR2 and TLR4 are the major TLRs mediating LPC function. LPC can induce the migration of lymphocytes and macrophages, upregulate pro-inflammatory cytokines, induce oxidative stress, and promote apoptosis, thereby aggravating inflammation and promoting the development of diseases [33,38]. Based on indirect changes in metabolites (LPC, LPE levels), we suggest that PLA2 regulates abnormal metabolic pathways through the TLR4/NF-κB axis, thereby inhibiting the inflammatory cascade. This idea still needs further verification.

Disrupted glycerophospholipid metabolism represents a key early lipid signature of inflammatory lung injury. In this study, serum levels of various lysophospholipids, including lysoPC, lysoPE, and PC, were significantly elevated, whereas LysoPC (20:4) containing an arachidonic acid (AA) side chain was markedly reduced in the model group compared to the control group (*p* < 0.05) (Table S2). These findings suggest enhanced PLA2 activation, increased membrane phospholipid hydrolysis, and rapid consumption of LysoPC (20:4) as a precursor, ultimately leading to substantial free AA release. Elevated free AA abundance serves as a substrate for synthesizing pro-inflammatory airway mediators, which exacerbate bronchial epithelial inflammation and airway hyperreactivity, aligning with lipid metabolic alterations observed in inflammatory lung injury [39,40]. Despite the expanded AA substrate pool, the lipoxygenase-derived metabolite 15(S)-HETE was significantly decreased in the model group (*p* < 0.01). This indicated a redirection of AA metabolic flux, characterized by selective inhibition of the 15-lipoxygenase (15-LOX) pathway and insufficient anti-inflammatory mediator production, while pro-inflammatory metabolic branches remained relatively overactive [41,42]. Prominent inflammatory cell infiltration in the lung sections of the model group further corroborated the dysregulated AA metabolism. Following SAE administration, serum AA levels were significantly reduced, while the 15(S)-HETE levels were significantly increased, with the BH group showing the most pronounced regulatory effect (Figure 7). These findings demonstrate that SAE inhibited excessive phospholipid hydrolysis, corrected imbalances in AA metabolic branching, and ameliorated lipid metabolic disorders and inflammatory damage in CSALI by restoring 15(S)-HETE biosynthesis. Other metabolites also contributed to CSALI progression. Indoleacrylic acid, a tryptophan metabolite, was significantly upregulated (*p* < 0.05) (Table S2). This metabolite modulates the aryl hydrocarbon receptor in airway epithelial cells, regulating lung innate immune homeostasis and participating in the inflammatory cascade of acute lung injury [43,44]. SAE treatment significantly decreased serum levels of these differential metabolites. Collectively, these results demonstrate that SAE exerts a distinct protective effect against CSALI by ameliorating metabolic disorders.

However, the CSALI model does not fully recapitulate the complex etiologies of human lung function decline and has not been validated at the tissue level by transcriptomic, proteomic, or key pathway-target analyses, thereby limiting in-depth investigation of the molecular mechanisms of the lung–liver axis. Moreover, the study was limited to male rats, which restricts the generalizability of the findings.

## 5. Conclusions

In this study, SAE suppressed inflammatory cell proliferation and migration, significantly attenuating inflammatory cell infiltration and ameliorating lung tissue damage in CSALI rats. It reduced serum levels of ALP, AST, ALT, TBIL, and DBIL and decreased levels of inflammatory cytokines, including TGF-β and IL-6, in both serum and BALF. Serum metabolomics analysis using ultra-performance liquid chromatography–high-resolution mass spectrometry (UPLC–HRMS) identified 24 differential metabolites between the control and model groups; these metabolites were enriched in AA metabolism and glycerophospholipid metabolism pathways implicated in CSALI pathogenesis. SAE treatment significantly modulated the levels of these differential metabolites. PLA2, a hypothetical mediator inferred from metabolomic data, may play a key role in this metabolic process. These findings provide a theoretical foundation for the development and application of SAE in the prevention of CSALI.

## Data Availability

The data that support the findings of this study are available on request from the corresponding author.

## Supplementary Materials

The following supporting information can be downloaded at https://www.mdpi.com/article/10.3390/metabo16070486/s1, Figure S1: Schematic diagram of animal experimental design (n = 8). Figure S2: PCA scores plots of rat serum samples (n = 8). Table S1: Cytotoxic evaluation of SAE in RAW264.7 cells. Table S2: Identification of 24 differential metabolites between the control group and the model group.

## Abbreviations

The following abbreviations are used in this manuscript:

AA	Arachidonic acid
ALP	Alkaline phosphatase
ALT	Alanine aminotransferase
AST	Aspartate aminotransferase
BALF	Bronchoalveolar lavage fluid
CCK-8	Cell counting kit-8
CSALI	Cold stress-aggravated acute lung injury
DBIL	Direct bilirubin
ESI	Electrospray ionization
H&E	Hematoxylin and eosin
IL-6	Interleukin-6
KEGG	Kyoto Encyclopedia of Genes and Genomes
lysoPC	Lysophosphatidylcholine
lysoPE	Lysophosphatidylethanolamine
LPS	Lipopolysaccharide
OPLS-DA	Orthogonal partial least squares-discriminant analysis
PC	Phosphatidylcholine
PCA	Principal component analysis
PLA2	Phospholipase A2
QC	Quality control
SAE	Star anise extract
TBIL	Total bilirubin
TCM	Traditional Chinese medicine
TGF-β	Transforming growth factor-β
TLRs	Toll-like receptors
VIP	Variable importance in the projection
