# Supplementary material for: Non-Targeted Metabolomics Profiling and Anti-Inflammatory Potential of Star Anise Extract in Rats with Cold Stress—Aggravated Acute Lung Injury
Source: Metabolites. 2026 Jul 10;16(7):486. doi: 10.3390/metabo16070486 (PMC13413549; doi:10.3390/metabo16070486)
Supplement: Supplementary file 1 [file metabolites-16-00486-s001.zip › metabolites-4318230-supplementary.pdf]

Table S1. Cytotoxic evaluation of SAE in RAW264.7 cells.

Cell viability (% of control) ($\bar{x}$ ± SD, n = 6)		
Dose/ (μg /mL)	24 h	48 h
0.1	118.38 ± 15.09*	112.35 ± 6.68
1	116.79 ± 6.00**	110.22 ± 15.87**
10	109.30 ± 6.45	96.94 ± 6.16
20	92.25 ± 14.43	66.59 ± 2.47*
50	84.19 ± 5.34*	27.73 ± 3.47**
100	11.92 ± 3.60**	2.08 ± 0.34**
200	5.84 ± 1.47**	1.62 ± 0.18**

Notes: *$P < 0.05$, **$P < 0.01$ *vs.* blank group. Cell viability was calculated and normalized with the untreated control group (without LPS simulation) set as 100%.

Table S2. Identification of 24 differential metabolites between the control group and the model group.

No.	Metabolite	Formula	RT (min)	*m/z*	Adducts	MS/MS fragments	M *vs.* C	SAE *vs.* M	Category	VIP
1	Cytidine	C9H13N3O5	0.63	242.0795	M-H	242.0783, 225.9858, 210.8399, 181.9647, 131.0805	↑ *	↓ #	Pyrimidine nucleosides	1.16
2	N-Acetylleucine	C8H15NO3	1.48	172.0977	M-H	172.0989, 153.1110, 130.0873, 110.0947, 84.8470	↑ *	↓ #	Carboxylic acids and derivatives	1.03
3	D-Tryptophan	C11H12N2O2	2.60	204.0892	M-H, 2M-H	203.0000, 187.8617, 168.8422, 157.8597,	↑ *	↓ #	Indoles and derivatives	2.51

						116.9292				
4	Indoleacrylic acid	C11H9NO2	2.62	188.0715	M+H	188.0690, 170.0589, 143.0725, 118.0649	↑ *	↓ #	Indoles and derivatives	3.04
5	3-Succinoylpyridine	C9H9NO3	3.37	178.0505	M-H	178.0507, 160.8422, 134.0623, 80.9622	↑ *	↓ #	Keto acids and derivatives	1.00
6	Corticosterone	C21H30O4	5.53	347.2229	M+H	347.2252, 317.2113, 299.2072, 161.0611,	↑ *	↓ #	Steroids and steroid derivatives	1.50
7	Taurochenodesoxycholic acid	C26H45NO6S	5.77	498.2884	M-H	498.2925, 480.2864, 400.3300, 374.2742, 304.1865, 106.9872	↑ **	↓ ##	Steroids and steroid derivatives	2.18
8	Sinapic acid	C11H12O5	6.24	447.1337	2M-H	447.1337, 223.0020, 207.1020, 179.1070, 162.9980	↓ *	↑ ##	Cinnamic acids and derivatives	1.01
9	Zingerone	C11H14O3	6.34	193.0869	M-H	193.0873, 177.0556, 135.9754, 121.0306, 93.0370	↑ **	↓ #	Phenols	1.06
10	Cervonoyl ethanolamide	C24H36O3	6.44	373.2741	M+H	373.2126, 329.1871, 199.1228, 133.0864	↑ *	↓ #	Fatty Acyls	3.56
11	12,13-DHOME	C18H34O4	7.18	313.2381	M-H	313.2409, 295.2274, 269.0031, 223.0020, 195.1440	↑ *	↓ ##	Fatty Acyls	2.70
12	LysoPE (0:0/18:0)	C23H48NO7P	7.38	526.3141	M+FA-H	526.3138, 480.2960, 419.0066, 180.9940	↑ *	↓ #	Glycerophospholipids	1.45
13	Tetracosahexaenoic acid	C24H36O2	7.52	357.2798	M+H	357.1709, 339.2025, 321.2516, 133.1012	↓ *	↑ #	Fatty Acyls	2.75
14	LysoPC (18:2(9Z,12Z))	C26H50NO7P	7.53	564.3302	M+FA-H	564.3281, 554.2991,	↑ *	↓ ##	Glycerophospholipids	3.25

						279.2340, 224.0663				
15	Arachidonic acid	C20H32O2	7.57	303.2329	M-H	303.2351, 285.2061, 259.2442, 219.1320, 179.1070, 139.0997	↑ **	↓ ##	Fatty Acyls	1.65
16	LysoPC (20:4(5Z,8Z,11Z,14Z))	C28H50NO7P	7.72	588.3302	M+FA-H	588.3256, 528.3094, 303.2351, 224.0663	↓ *	↑ #	Glycerophospholipids	2.69
17	LysoPE (18:1(11Z)/0:0)	C23H46NO7P	7.90	478.2955	M-H	478.2881, 460.2849, 281.0029, 152.9977	↑ *	↓ #	Glycerophospholipids	1.81
18	PC (16:0/20:1(11Z))	C44H86NO8P	7.99	810.6011	M+Na	810.5985, 788.6135, 605.3051, 496.3426	↑ *	↓ #	Glycerophospholipids	3.36
19	LysoPC (18:1(11Z))	C26H52NO7P	8.33	566.3457	M+FA-H	556.3163, 506.3285, 281.2519, 224.0729	↑ *	↓ ##	Glycerophospholipids	5.01
20	LysoPE (20:0/0:0)	C25H52NO7P	8.40	554.3441	M+FA-H	554.3505, 508.3505, 465.3114, 311.1651	↓ *	↑ #	Glycerophospholipids	1.02
21	15(S)-HETE	C20H32O3	8.59	320.235	M-H, 2M-H	319.2282, 301.2155, 257.2300, 179.1070, 116.9292	↓ **	↑ #	Fatty Acyls	8.82
22	Eicosapentaenoic acid	C20H30O2	8.60	302.2254	M+H, 2M+H	303.2335, 285.2264, 267.2136, 185.0734	↓ **	↑ #	Fatty Acyls	6.62
23	Ginkgoic acid	C22H34O3	8.72	345.2429	M-H	345.2462, 297.2419, 229.1812, 117.9278	↓ **	↑ #	Benzene and substituted derivatives	1.00
24	LysoPE (0:0/22:1(13Z))	C27H54NO7P	8.82	580.3616	M+FA-H	580.3656, 534.3495, 473.3344, 140.0180	↑ *	↓ ##	Glycerophospholipids	2.07

Note: *$P < 0.05$, **$P < 0.01$ compared to the control group; $^{\#}P < 0.05$, $^{\#\#}P < 0.01$ compared to the model group.

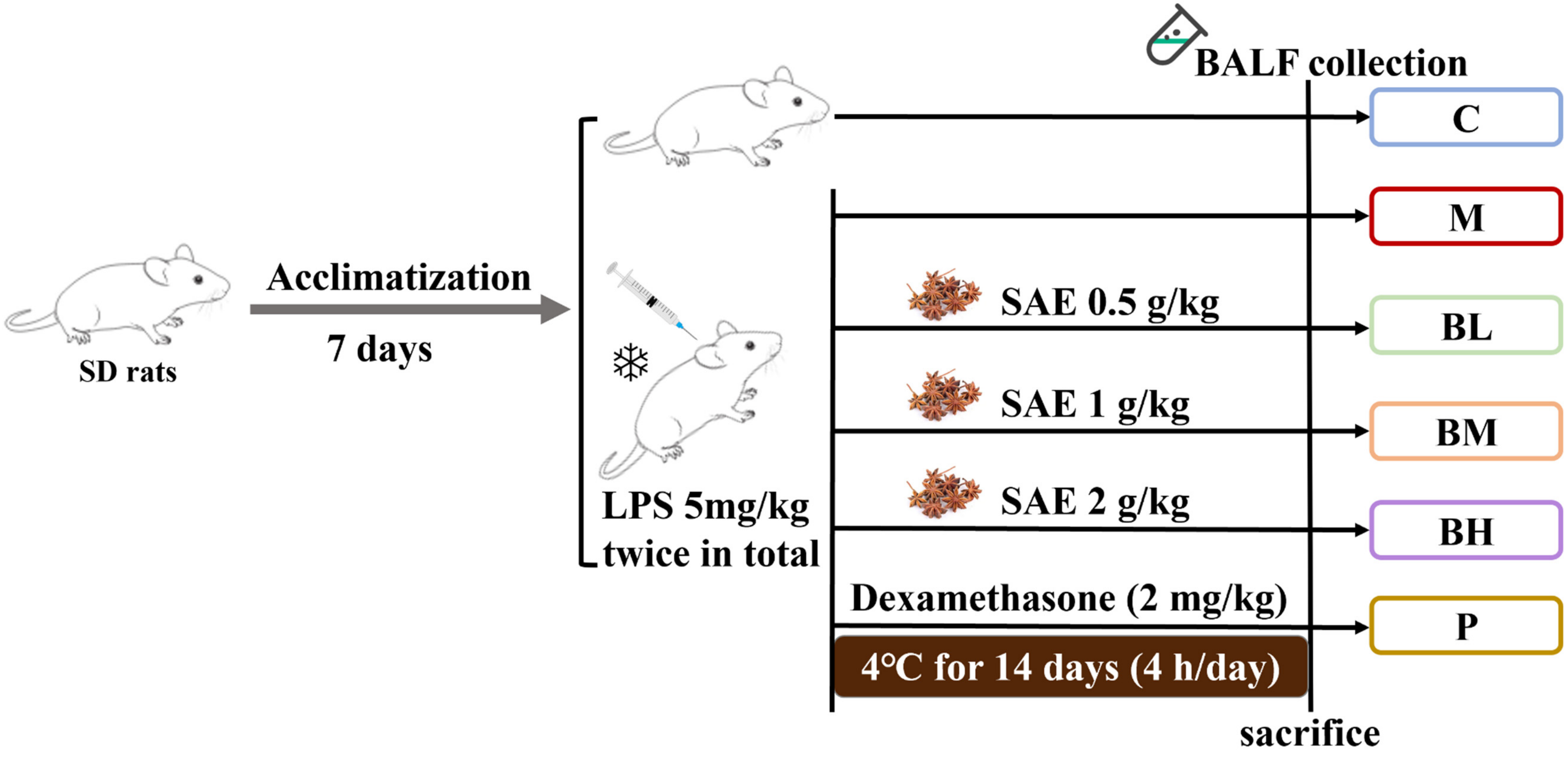

Figure S1. Schematic diagram of animal experimental design (n = 8). SAE, star anise extract; BALF, bronchoalveolar lavage fluid; LPS, lipopolysaccharide; C, the control group; M, the model group; BL, LPS+SAE low-dose group; BM, LPS+SAE medium-dose group; BH, LPS+SAE high-dose group.

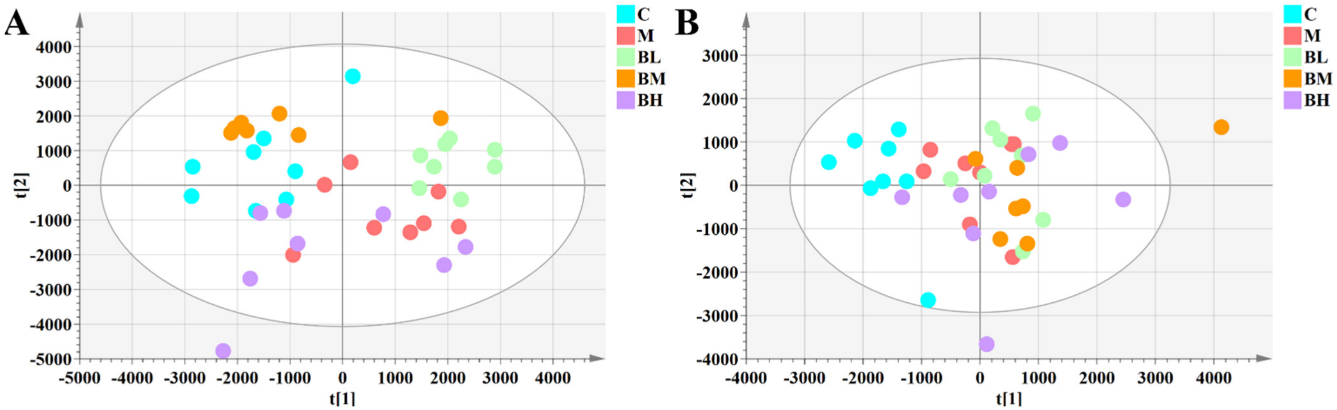

Figure S2. PCA scores plots of rat serum samples (n = 8). **(A)** Positive ion mode. **(B)** Negative ion mode.
